# Safety and tolerability of nintedanib in patients with progressive fibrosing interstitial lung diseases: data from the randomized controlled INBUILD trial

**DOI:** 10.1186/s12931-022-01974-2

**Published:** 2022-04-07

**Authors:** Vincent Cottin, Fernando J. Martinez, R. Gisli Jenkins, John A. Belperio, Hideya Kitamura, Maria Molina-Molina, Inga Tschoepe, Carl Coeck, Dirk Lievens, Ulrich Costabel

**Affiliations:** 1 National Reference Center for Rare Pulmonary Diseases, Louis Pradel Hospital, Hospices Civils de Lyon, Claude Bernard University Lyon 1, University of Lyon, IVPC, INRAE, ERN-LUNG, Lyon, France; 2 Weill Cornell Medicine, New York, NY USA; 3 National Heart and Lung Institute, Imperial College London, London, UK; 4 David Geffen School of Medicine at UCLA, Los Angeles, CA USA; 5 Department of Respiratory Medicine, Kanagawa Cardiovascular and Respiratory Center, Kanazawa-ku, Yokohama, Japan; 6 ILD Unit, University Hospital of Bellvitge, IDIBELL, Barcelona, Spain; 7 Elderbrook Solutions, Bietigheim-Bissingen, Germany; 8 SCS Boehringer Ingelheim Comm.V., Brussels, Belgium; 9 Boehringer Ingelheim International GmbH, Ingelheim am Rhein, Germany; 10 Center for Interstitial and Rare Lung Diseases, Ruhrlandklinik, University Hospital, University of Duisburg-Essen, Essen, Germany

**Keywords:** Adverse drug event, Clinical trial, Diarrhea, Patient adherence, Pulmonary fibrosis

## Abstract

**Background:**

In the INBUILD trial in patients with progressive fibrosing interstitial lung diseases (ILDs), nintedanib reduced the rate of decline in forced vital capacity compared with placebo, with side-effects that were manageable for most patients. We used data from the INBUILD trial to characterize further the safety and tolerability of nintedanib.

**Methods:**

Patients with fibrosing ILDs other than idiopathic pulmonary fibrosis (IPF), who had experienced progression of ILD within the 24 months before screening despite management deemed appropriate in clinical practice, were randomized to receive nintedanib 150 mg twice daily or placebo. To manage adverse events, treatment could be interrupted or the dose reduced to 100 mg twice daily. We assessed adverse events and dose adjustments over the whole trial.

**Results:**

A total of 332 patients received nintedanib and 331 received placebo. Median exposure to trial drug was 17.4 months in both treatment groups. Adverse events led to treatment discontinuation in 22.0% of patients treated with nintedanib and 14.5% of patients who received placebo. The most frequent adverse event was diarrhea, reported in 72.3% of patients in the nintedanib group and 25.7% of patients in the placebo group. Diarrhea led to treatment discontinuation in 6.3% of patients in the nintedanib group and 0.3% of the placebo group. In the nintedanib and placebo groups, respectively, 48.2% and 15.7% of patients had ≥ 1 dose reduction and/or treatment interruption. Serious adverse events were reported in 44.3% of patients in the nintedanib group and 49.5% of patients in the placebo group. The adverse event profile of nintedanib was generally consistent across subgroups based on age, sex, race and weight, but nausea, vomiting and dose reductions were more common among female than male patients.

**Conclusions:**

The adverse event profile of nintedanib in patients with progressive fibrosing ILDs other than IPF is consistent with its established safety and tolerability profile in patients with IPF and characterized mainly by gastrointestinal events, particularly diarrhea. Management of adverse events using symptomatic therapies and dose adjustment is important to minimize the impact of adverse events and help patients remain on therapy.

*Trial registration* Registered 21 December 2016, https://clinicaltrials.gov/ct2/show/NCT02999178

**Graphical Abstract:**

A video abstract summarizing the key results presented in this manuscript is available at: https://www.globalmedcomms.com/respiratory/cottin/INBUILDsafety.

**Supplementary Information:**

The online version contains supplementary material available at 10.1186/s12931-022-01974-2.

## Background

In addition to all patients with idiopathic pulmonary fibrosis (IPF), a proportion of patients with other chronic fibrosing interstitial lung diseases (ILDs) develop a progressive fibrosing phenotype characterized by increasing fibrosis on high resolution computed tomography (HRCT); worsening of lung function, symptoms and quality of life; and early mortality [1–4].

Nintedanib is an intracellular inhibitor of tyrosine kinases that inhibits processes fundamental to the progression of pulmonary fibrosis [5, 6]. Randomized placebo-controlled trials have demonstrated that nintedanib slows the progression of ILD in patients with IPF [7], systemic sclerosis-associated ILD (SSc-ILD) [8], and progressive fibrosing ILDs other than IPF [9]. The adverse event profile of nintedanib in patients with ILDs is characterized mainly by gastrointestinal adverse events, particularly diarrhea [7–13].

The INBUILD trial was conducted in patients with chronic fibrosing ILDs other than IPF that had progressed within the prior 24 months despite management deemed appropriate in clinical practice [9]. The trial population included patients with a variety of diagnoses who were taking a wide range of comedications [9, 14]. The primary endpoint of the annual rate of decline in forced vital capacity (FVC) was assessed over 52 weeks; however patients continued to receive blinded randomized treatment until the last patient had completed the trial, so most patients received trial medication for longer than 52 weeks. We used data from the whole INBUILD trial to perform a comprehensive analysis of the safety and tolerability of nintedanib in this patient population.

## Methods

### Trial design

The design of the INBUILD trial has been published, together with the trial protocol [9]. Briefly, patients had a physician-diagnosed chronic fibrosing ILD other than IPF, reticular abnormality with traction bronchiectasis (with or without honeycombing) of > 10% extent on HRCT, FVC ≥ 45% predicted, and diffusing capacity of the lung for carbon monoxide (DLco) ≥ 30% to < 80% predicted. Patients met one of the following criteria for ILD progression within the 24 months before screening, despite management deemed appropriate in clinical practice: relative decline in FVC ≥ 10% predicted; relative decline in FVC ≥ 5 to < 10% predicted and worsened respiratory symptoms; relative decline in FVC ≥ 5 to < 10% predicted and increased extent of fibrosis on HRCT; worsened respiratory symptoms and increased extent of fibrosis on HRCT. Patients taking azathioprine, cyclosporine, mycophenolate mofetil, tacrolimus, rituximab, cyclophosphamide, or oral glucocorticoids > 20 mg/day were not enrolled. Initiation of these medications was allowed after 6 months of the trial in cases of deterioration of ILD or autoimmune disease. Patients at risk of bleeding (defined as genetic predisposition to bleeding; requirement for fibrinolysis, full-dose therapeutic anticoagulation or high-dose antiplatelet therapy; hemorrhagic central nervous system event within 12 months; hemoptysis or hematuria, active gastrointestinal bleeding or gastrointestinal ulcers, or major injury or surgery, within 3 months; or international normalized ratio > 2, prolongation of prothrombin time and activated partial thromboplastin time by > 1.5 times the upper limit of the normal range (ULN) were not enrolled. Patients were excluded if they had a history of severe uncontrolled hypertension (≥ 160/100 mmHg) within 6 months, myocardial infarction within 6 months, unstable angina within 6 months, thrombotic event (including stroke and transient ischemic attack) within 12 months of screening, or significant pulmonary arterial hypertension.

Patients were randomized to receive nintedanib 150 mg twice daily (bid) or placebo, stratified by fibrotic pattern on HRCT (usual interstitial pneumonia [UIP]-like fibrotic pattern or other fibrotic patterns). Patients received one capsule bid administered orally. The trial consisted of two parts (Additional file 1). Part A comprised 52 weeks of treatment. Part B was a variable period beyond week 52 during which patients continued to receive blinded randomized treatment until all the patients had completed the first 52 weeks of the trial and the benefit-risk of nintedanib over 52 weeks had been assessed. Patients who discontinued treatment were asked to attend all visits as planned, including an end-of-treatment visit and a follow-up visit 4 weeks later. Patients who were still on treatment at the end of Part B were eligible to enter an open-label extension study, INBUILD-ON (NCT03820726). The final database lock took place after all patients had completed the follow-up visit or entered INBUILD-ON. Data collected up to the final database lock (i.e. in Part A and Part B) are referred to as data collected over the whole trial.

**Fig. 1:**
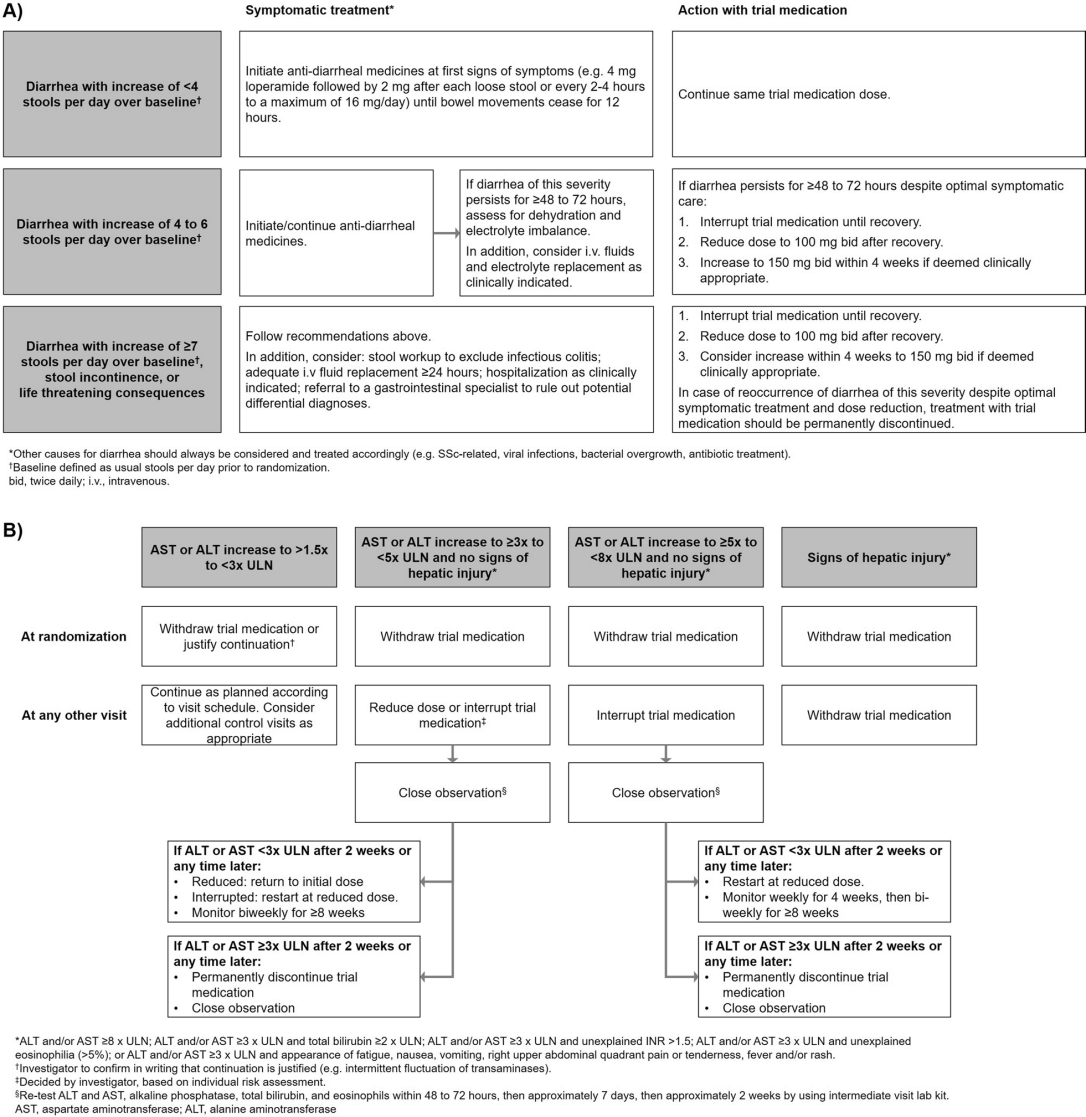
Algorithm for the management of **A** diarrhea adverse events and **B** hepatic enzyme elevations in the INBUILD trial

Treatment interruptions (for ≤ 4 weeks for adverse events considered related to trial medication or ≤ 8 weeks for other adverse events) and dose reductions to 100 mg bid were recommended to manage adverse events. No time frame was specified for the duration of dose reduction. After resolution of the adverse event, nintedanib could be reintroduced and/or the dose increased back to 150 mg bid. Specific recommendations were provided to the investigators for the management of diarrhea and hepatic enzyme elevations (Fig. 1). Adverse events were reported irrespective of causality and coded according to preferred terms in the Medical Dictionary for Regulatory Activities (MedDRA) version 22.0.

### Analyses

Compliance with trial medication was calculated as the number of capsules taken × 100 divided by the number of capsules that should have been taken. Dose intensity was defined as the amount of drug administered divided by the amount of drug that would have been received if the 150 mg bid dose had been administered for the planned treatment period or until permanent treatment discontinuation.

We analyzed the proportions of patients with any adverse events, any serious adverse events, and any adverse events leading to treatment discontinuation over 52 weeks (or until 28 days after the last intake of trial drug in patients who discontinued trial drug before week 52) and between the first trial drug intake and 28 days after the last trial drug intake (i.e. over the whole trial). Serious adverse events were defined as events that resulted in death, were life-threatening, resulted in hospitalization or prolongation of hospitalization, resulted in persistent or clinically significant disability or incapacity, were a congenital anomaly or birth defect, or were deemed to be serious for any other reason. The intensity of an adverse event was categorized by the investigator as mild (awareness of signs or symptoms which are easily tolerated), moderate (enough discomfort to cause interference with usual activity), or severe (incapacitating or causing inability to work or to perform usual activities). Based on the mechanism of action of nintedanib [5] and its known adverse event profile in patients with IPF [7], we present data on diarrhea, liver enzyme and bilirubin elevations, bleeding, and cardiovascular adverse events. Analyses were descriptive and based on patients who received ≥ 1 dose of trial drug.

## Results

### Patients

A total of 663 patients received ≥ 1 dose of trial drug (332 nintedanib, 331 placebo). The baseline characteristics of the trial population have been described [9, 14]. Briefly, mean (SD) age was 65.8 (9.8) years, FVC was 69.0 (15.6) % predicted; 53.7% of patients were male, 62.1% had a UIP-like fibrotic pattern on HRCT. The most common diagnoses were hypersensitivity pneumonitis (26.1%), autoimmune disease-related ILDs (25.6%), idiopathic non-specific interstitial pneumonia (iNSIP) (18.9%), and unclassifiable idiopathic interstitial pneumonia (17.2%). About half the patients (53.2%) were taking low-dose glucocorticoids (≤ 20 mg/day prednisone or equivalent), 4.7% were taking biologic disease-modifying antirheumatic drugs (DMARDs) and 11.6% were taking non-biologic DMARDs [14].

### Compliance and exposure

Over the whole trial, 34.3% of patients in the nintedanib group and 30.2% of patients in the placebo group permanently discontinued trial medication (for any reason). Among those who permanently discontinued medication in the nintedanib and placebo groups, respectively, 17.5% and 2.0% discontinued within 31 days; 15.8% and 12.0% discontinued between day 32 and day 91; 14.9% and 15.0% discontinued between day 92 and day 182; and 51.8% and 71.0% discontinued after day 182. Mean compliance with trial medication was 96.7% in the nintedanib group and 97.5% in the placebo group. Mean dose intensity was 91.5% and 98.4% in these groups, respectively. Median exposure to trial drug (at either dose) was 17.4 months in both treatment groups.

### Dose adjustments

Almost half (48.2%) of patients in the nintedanib group and 15.7% in the placebo group had ≥ 1 dose reduction and/or treatment interruption. The most common reason for dose reduction or treatment interruption was diarrhea (Tables 1 and 2). Information on dose reductions and treatment interruptions is shown in Tables 1 and 2. A total of 131 (39.5%) patients in the nintedanib group had ≥ 1 dose reduction, of whom 41 (31.3%) had ≥ 1 dose re-escalation. Twenty (6%) patients in the placebo group had ≥ 1 dose reduction, of whom 8 (40%) had ≥ 1 dose re-escalation. Dose reductions were more common among female patients (49.0% of the nintedanib group, 8.4% of the placebo group) than male patients (31.3% of the nintedanib group, 4.0% of the placebo group) (Additional file 2). A total of 128 (38.6%) patients in the nintedanib group and 41 (12.4%) patients in the placebo group had ≥ 1 treatment interruption.

**Table 1:** Dose reductions in the INBUILD trial

	Nintedanib (n = 332)	Placebo (n = 331)
Patients with ≥ 1 dose reduction	131 (39.5)	20 (6.0)
Number of dose reductions		
0	201 (60.5)	311 (94.0)
1	113 (34.0)	17 (5.1)
2	17 (5.1)	3 (0.9)
> 2	1 (0.3)	0
Total number of dose reductions	151	23
Time to first dose reduction (days)		
≤ 31	18 (5.4)	3 (0.9)
> 31 to ≤ 91	30 (9.0)	10 (3.0)
> 91 to ≤ 182	36 (10.8)	3 (0.9)
> 182	47 (14.2)	4 (1.2)
Most frequent reasons for dose reduction considered related to trial drug, n (%) of dose reductions^a^		
Diarrhea	68 (45.0)	3 (13.0)
ALT increased	16 (10.6)	3 (13.0)
Hepatic function abnormal	11 (7.3)	1 (4.3)
Nausea	10 (6.6)	2 (8.7)
Weight decreased	7 (4.6)	0
AST increased	6 (4.0)	0
Vomiting	6 (4.0)	1 (4.3)
Decreased appetite	4 (2.6)	1 (4.3)
Blood alkaline phosphatase increased	3 (2.0)	0
Liver function test increased	3 (2.0)	0

Data are n (%) of patients unless otherwise stated. Adverse events shown were reported between first trial drug intake and 28 days after last trial drug intake. Median exposure to trial drug was 17.4 months in both groups

*ALT* alanine aminotransferase, *AST* aspartate aminotransferase

^a^Adverse events were coded based on preferred terms in the Medical Dictionary for Regulatory Activities version 22.0. Adverse events that led to > 2 dose reductions in either treatment group are shown. Percentages are based on the total number of dose reductions

**Table 2:** Treatment interruptions in the INBUILD trial

	Nintedanib (n = 332)	Placebo (n = 331)
Patients with ≥ 1 treatment interruption	128 (38.6)	41 (12.4)
Number of treatment interruptions per patient		
0	204 (61.4)	290 (87.6)
1	81 (24.4)	32 (9.7)
2	30 (9.0)	7 (2.1)
> 2	17 (5.1)	2 (0.6)
Total number of treatment interruptions	197	52
Time to first treatment interruption (days)		
≤ 31	21 (6.3)	8 (2.4)
> 31 to ≤ 91	33 (9.9)	9 (2.7)
> 91 to ≤ 182	27 (8.1)	7 (2.1)
> 182	47 (14.2)	17 (5.1)
Total duration of treatment interruptions (days), mean (SD)	25.7 (20.1)	24.1 (22.0)
Most frequent reasons for treatment interruption considered related to trial drug, n (%) of interruptions^a^		
Diarrhea	73 (37.1)	5 (9.6)
ALT increased	14 (7.1)	1 (1.9)
Hepatic function abnormal	10 (5.1)	1 (1.9)
AST increased	7 (3.6)	0
Vomiting	7 (3.6)	1 (1.9)
Nausea	6 (3.0)	1 (1.9)

Data are n (%) of patients unless otherwise stated. Adverse events shown were reported between first trial drug intake and 28 days after last trial drug intake. Median exposure to trial drug was 17.4 months in both groups

*ALT* alanine aminotransferase, *AST* aspartate aminotransferase

^a^Adverse events were coded based on preferred terms in the Medical Dictionary for Regulatory Activities version 22.0. Adverse events that led to > 2 treatment interruptions in either treatment group are shown. Percentages are based on the total number of treatment interruptions

**Fig. 2:**
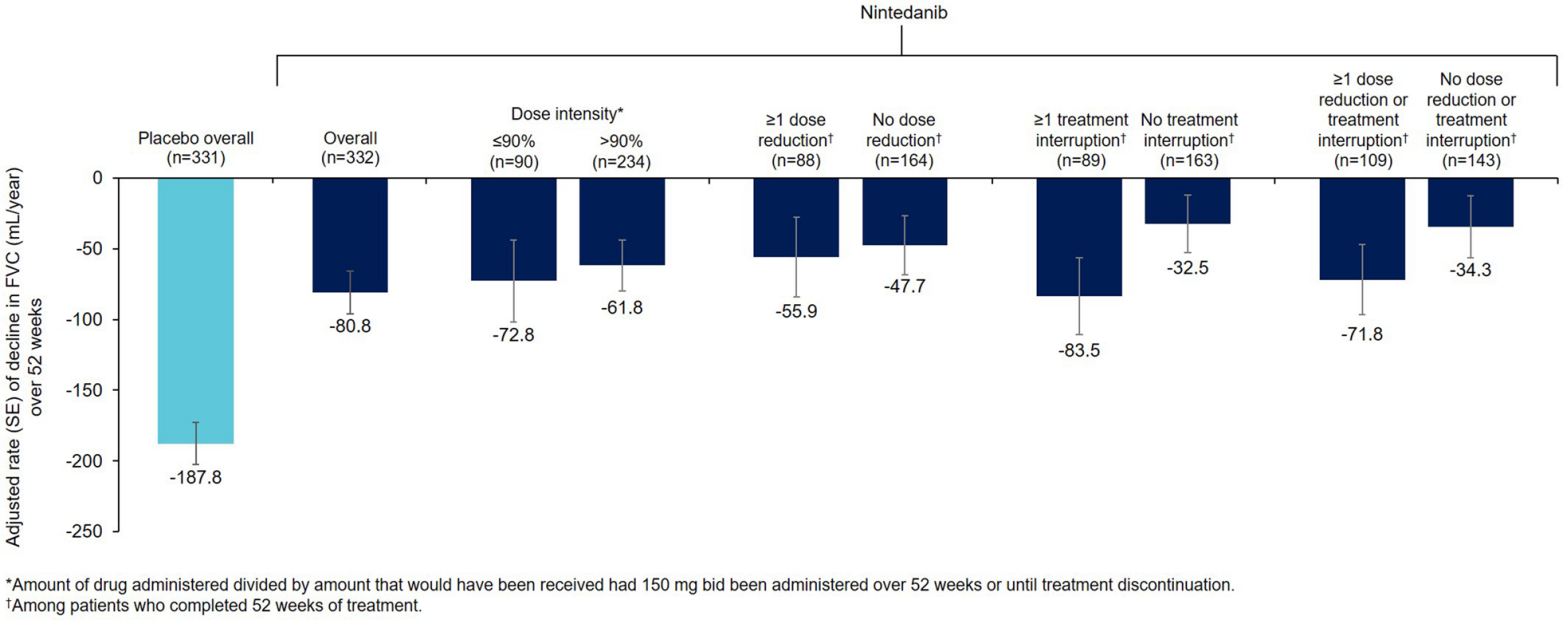
Rate of decline in FVC (mL/year) over 52 weeks by nintedanib dose adjustment in the INBUILD trial

Dose adjustments and treatment discontinuations over 52 weeks are shown in Additional file 3. In patients who completed 52 weeks of treatment, the rate of decline in FVC over 52 weeks was similar irrespective of the dose adjustments used to manage adverse events of nintedanib (Fig. 2).

### Adverse events

Adverse events over 52 weeks are described in Additional file 4. Over the whole trial, similar proportions of patients in the nintedanib and placebo groups had any adverse event(s) (98.2% and 93.1%, respectively). The most common adverse event was diarrhea, which was reported in 72.3% of patients in the nintedanib group and 25.7% of patients in the placebo group. Diarrhea occurred at a rate of 136.4 events per 100 patient-years in the nintedanib group and 23.0 events per 100 patient-years in the placebo group (Table 3). Adverse events in subgroups by sex, age, race and weight at baseline are shown in Additional file 5.

**Table 3:** Most frequent adverse events (reported irrespective of causality) in the INBUILD trial

	Nintedanib (n = 332)		Placebo (n = 331)	
	n (%)	Rate per 100 patient-years	n (%)	Rate per 100 patient-years
Diarrhea	240 (72.3)	136.4	85 (25.7)	23.0
Nausea	100 (30.1)	30.8	33 (10.0)	7.6
Vomiting	64 (19.3)	17.3	16 (4.8)	3.5
Abdominal pain	62 (18.7)	16.7	19 (5.7)	4.2
Nasopharyngitis	54 (16.3)	13.9	48 (14.5)	11.4
Decreased appetite	54 (16.3)	14.0	23 (6.9)	5.1
Dyspnea	52 (15.7)	12.9	57 (17.2)	13.3
Bronchitis	48 (14.5)	12.1	64 (19.3)	15.4
Weight decreased	49 (14.8)	12.4	18 (5.4)	3.9
ALT increased	49 (14.8)	12.4	13 (3.9)	2.8
AST increased	43 (13.0)	10.8	13 (3.9)	2.8
Cough	40 (12.0)	9.8	51 (15.4)	12.1
Progression of ILD^a^	28 (8.4)	6.5	56 (16.9)	12.7

Data are based on adverse events reported between first trial drug intake and 28 days after last trial drug intake. Median exposure to trial drug was 17.4 months in both groups. Adverse events were coded based on single preferred terms in the Medical Dictionary for Regulatory Activities (MedDRA) version 22.0, except for abdominal pain, which was based on a group of MedDRA preferred terms. Adverse events with a rate > 10 events per 100 patient-years in either treatment group are shown

*ALT* alanine aminotransferase, *AST* aspartate aminotransferase

^a^Based on MedDRA preferred term “interstitial lung disease”

Adverse events leading to treatment discontinuation occurred in a greater proportion of patients in the nintedanib group than in the placebo group (22.0% versus 14.5%) (Table 4). The rate of adverse events leading to treatment discontinuation was 17.0 events per 100 patient-years in the nintedanib group and 10.3 events per 100 patient-years in the placebo group. Diarrhea led to permanent treatment discontinuation in 6.3% of patients in the nintedanib group and 0.3% of patients who received placebo.

**Table 4:** Most frequent adverse events that led to permanent treatment discontinuation in the INBUILD trial

	Nintedanib (n = 332)		Placebo (n = 331)	
	n (%)	Rate per 100 patient-years	n (%)	Rate per 100 patient-years
Any adverse event(s) leading to permanent treatment discontinuation	73 (22.0)	17.0	48 (14.5)	10.3
Diarrhea	21 (6.3)	4.8	1 (0.3)	0.2
ALT increased	6 (1.8)	1.4	1 (0.3)	0.2
Drug-induced liver injury	5 (1.5)	1.1	0	0
Progression of ILD^a^	3 (0.9)	0.7	12 (3.6)	2.6

Data are based on adverse events reported between first trial drug intake and 28 days after last trial drug intake. Median exposure to trial drug was 17.4 months in both groups. Adverse events were coded based on preferred terms in the Medical Dictionary for Regulatory Activities (MedDRA) version 22.0. Adverse events that led to permanent treatment discontinuation with an incidence rate of > 1 event per 100 patient-years in either group are shown

*ALT* alanine aminotransferase

^a^Based on MedDRA preferred term “interstitial lung disease”

Serious adverse events were reported in 44.3% of patients in the nintedanib group and 49.5% of patients in the placebo group. The most common serious adverse events in the nintedanib and placebo groups, respectively, were pneumonia (7.2% and 4.8%) and progression of ILD (5.7% and 13.6%) (Additional file 6). The proportion of patients with fatal adverse events in the nintedanib group (6.3%) was lower than that in the placebo group (10.9%).

### Gastrointestinal and metabolic adverse events

Diarrhea, nausea, vomiting, decreased appetite, weight decrease, and abdominal pain were more frequent in patients who received nintedanib than placebo (Table 3). Among nintedanib-treated patients who experienced ≥ 1 diarrhea adverse event, most (87.8%) experienced events that were at worst of mild or moderate intensity (Table 5). Among nintedanib-treated patients who experienced ≥ 1 diarrhea adverse event, 8.8% permanently discontinued treatment due to diarrhea.

**Table 5:** Number, intensity and consequences for trial drug of diarrhea adverse events among patients who experienced ≥ 1 diarrhea adverse event in the INBUILD trial

	Nintedanib (n = 238)	Placebo (n = 86)
Number of diarrhea events		
1	112 (47.1)	70 (81.4)
2	46 (19.3)	11 (12.8)
3	42 (17.6)	2 (2.3)
≥ 4	38 (16.0)	3 (3.5)
Time to onset of first diarrhea event (days)		
≤ 31	114 (47.9)	41 (47.7)
> 31 to ≤ 61	22 (9.2)	13 (15.1)
> 61 to ≤ 91	21 (8.8)	6 (7.0)
> 91 to ≤ 182	35 (14.7)	8 (9.3)
> 182	46 (19.3)	18 (20.9)
CTCAE grade of worst diarrhea event		
1 (mild)	149 (62.6)	70 (81.4)
2 (moderate)	60 (25.2)	10 (11.6)
3 (severe)	29 (12.2)	6 (7.0)
≥ 4 (life-threatening/fatal)	0	0
Worst consequence of diarrhea event for trial drug^a^		
Dose reduction	59 (24.8)	3 (3.5)
Discontinued trial drug	21 (8.8)	1 (1.2)
Neither of above	158 (66.4)	82 (95.3)

CTCAE: Common Terminology Criteria for Adverse Events [26]. Adverse events reported between first trial drug intake and 28 days after last trial drug intake are shown. Median exposure to trial drug was 17.4 months in both groups. Data are n (% of patients who had ≥ 1 diarrhea adverse event and in whom additional information was collected)

^a^Discontinuation of trial drug was considered the worst consequence, followed by dose reduction

### Hepatic adverse events

The proportions of patients with hepatic adverse events and elevations in hepatic enzymes and bilirubin were greater in patients treated with nintedanib than placebo (Table 6). Elevations in alanine aminotransferase (ALT) and/or aspartate aminotransferase (AST) to ≥ 3 times the ULN were observed in 47 patients (14.2%) in the nintedanib group and 6 patients (1.8%) in the placebo group. Among the 47 nintedanib-treated patients with these elevations, the elevations were observed ≤ 30 days after the start of treatment in 25 patients (53.2%), > 30 to ≤ 60 days after the start of treatment in 3 patients (6.4%), > 60 to ≤ 90 days after the start of treatment in 5 patients (10.6%) and > 90 days after the start of treatment in 14 patients (29.8%). For most of these cases (43 of 47 patients in the nintedanib group, 6 of 6 patients in the placebo group), liver enzymes returned to within the normal range spontaneously or after dose reduction or treatment interruption. One patient in each treatment group had elevations in liver enzymes and bilirubin that met criteria for Hy’s law; liver enzymes and bilirubin returned to normal ranges after discontinuation of nintedanib or placebo.

**Table 6:** Hepatic adverse events and elevations in liver enzymes and bilirubin in the INBUILD trial

	Nintedanib (n = 332)	Placebo (n = 331)
Hepatic adverse event^a^	87 (26.2)	24 (7.3)
Elevations in ALT and/or AST		
≥ 3 × ULN	47 (14.2)	6 (1.8)
≥ 5 × ULN	14 (4.2)	1 (0.3)
≥ 8 × ULN	3 (0.9)	1 (0.3)
Elevations in ALT and/or AST ≥ 3 × ULN and bilirubin ≥ 2 × ULN^b^	0 (0)	1 (0.3)
Elevation in total bilirubin		
≥ 1.5 × ULN	3 (0.9)	6 (1.8)
≥ 2 × ULN	1 (0.3)	1 (0.3)
Elevation in alkaline phosphatase		
≥ 1.5 × ULN	17 (5.1)	6 (1.8)
≥ 2 × ULN	8 (2.4)	3 (0.9)

Data are n (%) of patients with elevations reported between first trial drug intake and 28 days after last trial drug intake. Median exposure to trial drug was 17.4 months in both groups. Liver enzyme and bilirubin elevations are based on central laboratory data

*ALT* alanine aminotransferase, *AST* aspartate aminotransferase, *ULN* upper limit of normal

^a^Based on the standardized MedDRA query ‘liver related investigations, signs and symptoms’ (broad definition) which included preferred terms such as “ALT increased”, “AST increased” and “gamma-glutamyltransferase increased”

^b^One patient in the nintedanib group had elevations in ALT and/or AST ≥ 3 × ULN and bilirubin ≥ 2 × ULN based on local laboratory data. One patient in each group met criteria for Hy’s Law

### Bleeding adverse events

Bleeding adverse events were reported in 13.9% patients in the nintedanib group and 15.4% of patients in the placebo group (Additional file 7). The most frequent bleeding adverse event was non-serious epistaxis. One patient in each treatment group had a non-serious adverse event of gastrointestinal perforation. Serious bleeding adverse events occurred in 1.5% and 2.1% of patients in the nintedanib and placebo groups, respectively (Additional file 7). The most frequent serious bleeding adverse event in the nintedanib group was gastrointestinal hemorrhage (0.9% of patients).

### Cardiovascular adverse events

In the nintedanib and placebo groups, respectively, hypertension was reported in 5.7% and 6.9% of patients, major adverse cardiovascular events in 6.3% and 5.1% of patients, and myocardial infarction in 1.8% and 1.5% of patients (Additional file 8).

## Discussion

We used data from the INBUILD trial to characterize the safety and tolerability of nintedanib in patients with progressive fibrosing ILDs other than IPF. The adverse event profile of nintedanib in these patients was consistent with that observed in patients with IPF in the INPULSIS trials [7, 10] and in patients with SSc-ILD in the SENSCIS trial [8, 12].

Consistent with the findings of the INPULSIS and SENSCIS trials [10, 12], diarrhea was the most common adverse event associated with nintedanib and was mild or moderate in most cases. Serious adverse events of diarrhea were rare. Fewer than 9% of nintedanib-treated patients who experienced diarrhea discontinued nintedanib due to this adverse event. The low rate of discontinuation of nintedanib due to diarrhea or other gastrointestinal adverse events suggests that the recommendations provided for management of these events were effective in minimizing their impact and enabling patients to remain on treatment, and that similar procedures should be implemented in clinical practice.

The proportion of patients who permanently discontinued nintedanib over 52 weeks was similar in the INBUILD trial (24.1%) and INPULSIS trials (24.5%) and higher than in the SENSCIS trial (19.4%). The proportion of nintedanib-treated patients who had at least one dose reduction over 52 weeks was similar in the INBUILD trial (33.7%) and INPULSIS trials (27.9%) and lower than in the SENSCIS trial (40.6%). The reasons for this greater use of dose reduction are not known, but may be partly due to the greater proportion of female patients in the SENSCIS trial (75.2%) compared with the INBUILD trial (46.3%) or INPULSIS trials (20.7%), as dose reductions were more common in female than male patients. Among patients who completed 52 weeks of treatment, the rate of decline in FVC was similar irrespective of nintedanib dose adjustments. Despite this observation, we believe, based on a dose-finding study in patients with IPF, that a 150 mg bid dose of nintedanib is the optimal dose for slowing decline in FVC [15]. Other than in patients with mild hepatic impairment, nintedanib should be initiated at this dose to ensure that patients receive the optimal benefit on slowing progression of ILD.

Previous analyses of data from the INBUILD trial have shown that the adverse event profile of nintedanib was consistent across subgroups based on ILD diagnosis [16]. Here we showed that the adverse event profile of nintedanib was generally consistent across subgroups based on age, sex, race and weight, but nausea, vomiting and dose reductions were numerically more common among female than male patients. In the nintedanib group, ALT/AST increases were more frequent among patients who were female, Asian, or < 65 kg. The frequency of liver enzyme elevations in both the nintedanib and placebo groups was higher in the INBUILD trial than in the INPULSIS trials [7, 10] or SENSCIS trial [8, 12]. No reasons for this could be identified based on the characteristics of the patient populations, including sex, race, weight and concomitant medication use, and it is likely largely explained by the use of a different central laboratory, which used different reference values, i.e., a lower threshold for the upper limit of normal, in the INBUILD trial than in previous trials. In most cases, elevated liver enzymes returned to within the normal range spontaneously or after dose reduction or treatment interruption. Liver function tests should be conducted prior to initiation of nintedanib, at regular intervals during the first 3 months of treatment, and periodically thereafter or as clinically indicated [17].

Immunomodulatory therapies are the mainstay of treatment for fibrosing ILDs other than IPF [18, 19]. At baseline, glucocorticoids were taken by over half the patients in the INBUILD trial. To minimize the potential impact of immunomodulatory therapies on the assessment of the efficacy and safety of nintedanib, the use of certain immunomodulatory therapies was restricted. Use of nintedanib (other than as trial medication) and pirfenidone was prohibited. Previous analyses have shown that the adverse event profile of nintedanib over 52 weeks, including hepatic enzyme elevations, was similar between patients who did (n = 39) and did not (n = 293) use prohibited or restricted therapies at baseline or during treatment with trial drug [14] and between patients who did (n = 187) and did not (n = 145) use DMARDs and/or glucocorticoids at baseline [20]. A limitation of our data is that it was not possible to draw conclusions about the safety and tolerability of nintedanib used in combination with specific other medications, given the small numbers of patients using specific therapies and the differences between patients who were and were not taking those therapies.

As an inhibitor of the vascular endothelial growth factor receptor, nintedanib may increase the risk of bleeding [21]. In the INBUILD trial, bleeding adverse events and serious bleeding adverse events were not more frequent in patients treated with nintedanib than placebo, but it must be borne in mind that patients treated with full-dose anticoagulation, high-dose antiplatelet therapy or at high risk of bleeding were excluded. Pharmacovigilance data and other real-world evidence have shown that most bleeding events reported in patients with IPF treated with nintedanib were non-serious and that epistaxis and contusion were the most common bleeding events [11, 22]. It is recommended that patients treated with nintedanib who are on full-dose anticoagulation therapy be monitored closely for bleeding and anticoagulation treatment be adjusted as necessary [17].

Overall, the frequency of cardiovascular adverse events in the INBUILD trial was low. The proportions of patients with major adverse cardiovascular events or myocardial infarction were similar in the nintedanib and placebo groups. Data from clinical trials in patients with IPF have also shown that the incidence of cardiovascular events in patients treated with nintedanib is low and similar to that in patients who received placebo [23]. It should be noted that patients with a recent history of myocardial infarction, unstable angina or stroke were excluded from clinical trials of nintedanib.

The open-label extensions of the INPULSIS and SENSCIS trials, INPULSIS-ON and SENSCIS-ON, suggest that the safety and tolerability of nintedanib in patients with IPF and systemic sclerosis are maintained with long-term use [24, 25]. Data from the open-label extension of the INBUILD trial, INBUILD-ON, as well as from registries and other real-world studies, will provide further information on the long-term safety and tolerability of nintedanib in patients with ILDs.

## Conclusions

In conclusion, data from the INBUILD trial demonstrate that the adverse event profile of nintedanib in patients with progressive fibrosing ILDs other than IPF is consistent with its established safety and tolerability profile in patients with IPF and SSc-ILD. The adverse event profile of nintedanib is characterized mainly by gastrointestinal events, particularly diarrhea. Management of adverse events using symptomatic therapies and dose adjustment is important to minimize the impact of adverse events and help patients remain on therapy.

## Supplementary Information

**Additional file 1: Figure S1.** Design of the INBUILD trial (A) and time in the INBUILD trial on subject level (B).

**Additional file 2: Table S1.** Dose reductions and treatment interruptions in the INBUILD trial in subgroups by sex.

**Additional file 3: Table S2.** Dose reductions, treatment interruptions and exposure over 52 weeks in the INBUILD trial.

**Additional file 4: Table S3.** Adverse events over 52 weeks of the INBUILD trial.

**Additional file 5: Table S4.** Most frequent adverse events in the INBUILD trial in subgroups by sex. **Table S5.** Most frequent adverse events in the INBUILD trial in subgroups by age at baseline. **Table S6.** Most frequent adverse events in the INBUILD trial in subgroups by race. **Table S7.** Most frequent adverse events in the INBUILD trial in subgroups by weight at baseline.

**Additional file 6: Table S8.** Most frequent serious adverse events in the INBUILD trial.

**Additional file 7: Table S9.** Bleeding adverse events in the INBUILD trial.

**Additional file 8: Table S10.** Cardiovascular events in the INBUILD trial.

## Data Availability

To ensure independent interpretation of clinical study results, BI grants all external authors access to relevant material, including participant-level clinical study data, as needed to fulfil their role and obligations as authors under the ICMJE criteria. Clinical study documents and participant clinical study data are available on request after publication of the primary manuscript in a peer-reviewed journal, and if regulatory activities are complete and other criteria met as per the BI Policy on Transparency and Publication of Clinical Study Data (https://www.mystudywindow.com/msw/datasharing). Bona fide, qualified scientific and medical researchers are eligible to request access to the clinical study data with corresponding documentation describing the structure and content of the datasets. Upon approval, and governed by a Legal Agreement, data are shared in a secured data-access system for a period of 1 year, which may be extended upon request. Prior to providing access, clinical study documents and data will be examined, and, if necessary, redacted and de-identified, to protect the personal data of study participants and personnel, and to respect the boundaries of informed consent. Researchers should use the https://vivli.org/ link to request access to study data and visit https://www.mystudywindow.com/msw/datasharing for further information.

## Abbreviations

ALT: Alanine aminotransferase
AST: Aspartate aminotransferase
bid: Twice daily
CTCAE: Common Terminology Criteria for Adverse Events
DLco: Diffusing capacity of the lungs for carbon monoxide
DMARDs: Disease-modifying antirheumatic drugs
FVC: Forced vital capacity
HRCT: High resolution computed tomography
ILD: Interstitial lung disease
iNSIP: Idiopathic non-specific interstitial pneumonia
IPF: Idiopathic pulmonary fibrosis
MedDRA: Medical Dictionary for Regulatory Activities
SSc-ILD: Systemic sclerosis-associated interstitial lung disease
UIP: Usual interstitial pneumonia
ULN: Upper limit of the normal range
