# Supplementary material for: Safety and tolerability of nintedanib in patients with progressive fibrosing interstitial lung diseases: data from the randomized controlled INBUILD trial
Source: Respir Res. 2022 Apr 7;23:85. doi: 10.1186/s12931-022-01974-2 (PMC8991727; doi:10.1186/s12931-022-01974-2)

**Additional file 1: Figure S1**

Design of the INBUILD trial (A) and time in the INBUILD trial on subject level (B). Adapted from N Engl J Med, Flaherty KR et al, Nintedanib in progressive fibrosing interstitial lung diseases, Volume 381, 1718–27. Copyright © (2019) Massachusetts Medical Society. Reprinted with permission from Massachusetts Medical Society.

**A**

**
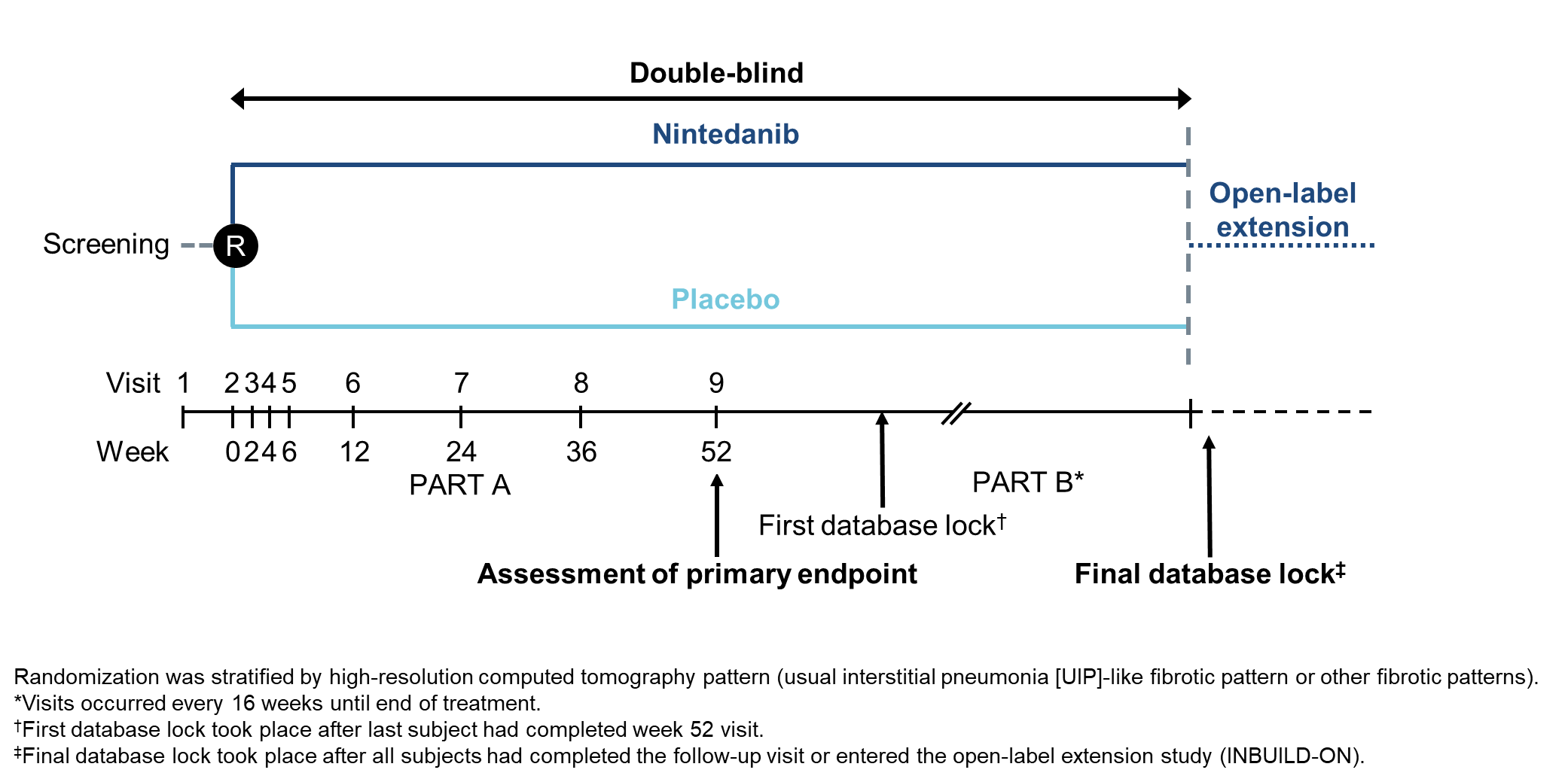
**

**B**


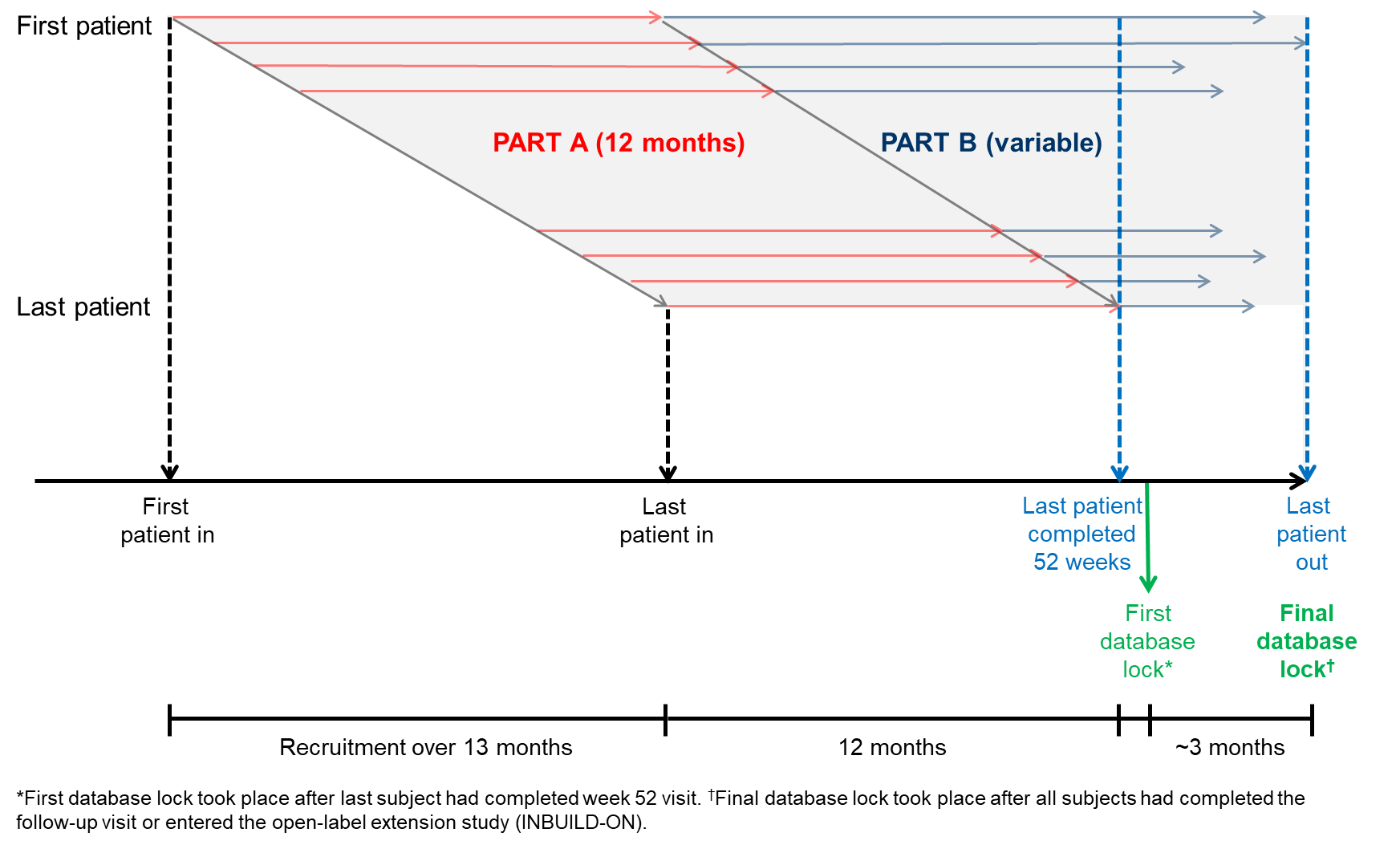

Supplement: Supplementary file 1 — Additional file 1: Figure S1. Design of the INBUILD trial (A) and time in the INBUILD trial on subject level (B). [file 12931_2022_1974_MOESM1_ESM.docx]
