# Supplementary material for: Safety and tolerability of nintedanib in patients with progressive fibrosing interstitial lung diseases: data from the randomized controlled INBUILD trial
Source: Respir Res. 2022 Apr 7;23:85. doi: 10.1186/s12931-022-01974-2 (PMC8991727; doi:10.1186/s12931-022-01974-2)
Supplement: Supplementary file 2 — Additional file 2: Table S1. Dose reductions and treatment interruptions in the INBUILD trial in subgroups by sex. [file 12931_2022_1974_MOESM2_ESM.docx]

**Additional file 2: Table S1**

Dose reductions and treatment interruptions in the INBUILD trial in subgroups by sex.

|  | **Male** | | **Female** | |
| --- | --- | --- | --- | --- |
|  | **Nintedanib (n=179)** | **Placebo (n=177)** | **Nintedanib (n=153)** | **Placebo (n=154)** |
| ≥1 dose reduction | 56 (31.3) | 7 (4.0) | 75 (49.0) | 13 (8.4) |
| Number of dose reductions |  |  |  |  |
| 0 | 123 (68.7) | 170 (96.0) | 78 (51.0) | 141 (91.6) |
| 1 | 49 (27.4) | 7 (4.0) | 64 (41.8) | 10 (6.5) |
| 2 | 6 (3.4) | 0 | 11 (7.2) | 3 (1.9) |
| >2 | 1 (0.6) | 0 | 0 | 0 |
| ≥1 treatment interruption | 67 (37.4) | 23 (13.0) | 61 (39.9) | 18 (11.7) |
| Number of treatment interruptions |  |  |  |  |
| 0 | 112 (62.6) | 154 (87.0) | 92 (60.1) | 136 (88.3) |
| 1 | 42 (23.5) | 20 (11.3) | 39 (25.5) | 12 (7.8) |
| 2 | 15 (8.4) | 3 (1.7) | 15 (9.8) | 4 (2.6) |
| >2 | 10 (5.6) | 0 | 7 (4.6) | 2 (1.3) |

Data are n (%) of patients. Dose reductions and treatment interruptions between first and last trial drug intake are shown.
