# Supplementary material for: Safety and tolerability of nintedanib in patients with progressive fibrosing interstitial lung diseases: data from the randomized controlled INBUILD trial
Source: Respir Res. 2022 Apr 7;23:85. doi: 10.1186/s12931-022-01974-2 (PMC8991727; doi:10.1186/s12931-022-01974-2)
Supplement: Supplementary file 3 — Additional file 3: Table S2. Dose reductions, treatment interruptions and exposure over 52 weeks in the INBUILD trial. [file 12931_2022_1974_MOESM3_ESM.docx]

**Additional file 3: Table S2**

Dose reductions, treatment interruptions and exposure over 52 weeks in the INBUILD trial.

|  | **Nintedanib  (n=332)** | **Placebo (n=331)** |
| --- | --- | --- |
| Patients who permanently discontinued trial medication | 84 (25.3) | 57 (17.2) |
| Patients with ≥1 dose reduction | 112 (33.7) | 18 (5.4) |
| Number of dose reductions per patient |  |  |
| 1 | 98 (29.5) | 16 (4.8) |
| 2 | 14 (4.2) | 2 (0.6) |
| Patients with ≥1 treatment interruption | 110 (33.1) | 34 (10.3) |
| Number of interruptions per patient |  |  |
| 1 | 74 (22.3) | 28 (8.5) |
| 2 | 29 (8.7) | 5 (1.5) |
| >2 | 7 (2.1) | 1 (0.3) |
| Total duration of interruption, days, median (minimum, maximum) | 16.5 (1, 72) | 12.0 (1, 66) |
| Median (min, max) exposure, months | 12.2 (0.0, 12.2) | 12.2 (0.3, 12.2) |
| Dose intensity*, %, mean (SD) | 92.5 (11.9) | 98.6 (5.4) |

Data are n (%) of patients unless otherwise stated. *Amount of drug administered divided by the amount of drug that would have been received if the 150 mg bid dose had been administered during the planned treatment period, or until permanent treatment discontinuation.
