# Supplementary material for: Safety and tolerability of nintedanib in patients with progressive fibrosing interstitial lung diseases: data from the randomized controlled INBUILD trial
Source: Respir Res. 2022 Apr 7;23:85. doi: 10.1186/s12931-022-01974-2 (PMC8991727; doi:10.1186/s12931-022-01974-2)
Supplement: Supplementary file 4 — Additional file 4: Table S3. Adverse events over 52 weeks of the INBUILD trial. [file 12931_2022_1974_MOESM4_ESM.docx]

**Additional file 4: Table S3**

Adverse events over 52 weeks of the INBUILD trial.

|  | **Nintedanib (n=332)** | **Placebo (n=331)** |
| --- | --- | --- |
| Any adverse event | 317 (95.5) | 296 (89.4) |
| Most frequent adverse events* |  |  |
| Diarrhea | 222 (66.9) | 79 (23.9) |
| Nausea | 96 (28.9) | 31 (9.4) |
| Bronchitis | 41 (12.3) | 47 (14.2) |
| Nasopharyngitis | 44 (13.3) | 40 (12.1) |
| Dyspnea | 36 (10.8) | 44 (13.3) |
| Vomiting | 61 (18.4) | 17 (5.1) |
| Cough | 33 (9.9) | 44 (13.3) |
| Abdominal pain | 60 (18.1) | 16 (4.8) |
| Decreased appetite | 48 (14.5) | 17 (5.1) |
| Headache | 35 (10.5) | 23 (6.9) |
| ALT increased | 43 (13.0) | 12 (3.6) |
| Progression of ILD^†^ | 16 (4.8) | 39 (11.8) |
| Weight decreased | 41 (12.3) | 11 (3.3) |
| AST increased | 38 (11.4) | 12 (3.6) |
| Serious adverse event^‡^ | 107 (32.2) | 110 (33.2) |
| Fatal adverse event | 11 (3.3) | 17 (5.1) |
| Adverse event leading to permanent treatment discontinuation | 65 (19.6) | 34 (10.3) |

Data are based on adverse events reported over 52 weeks (or until 28 days after last trial drug intake in patients who discontinued trial drug before week 52). Data are n (%) of patients with ≥1 such adverse event reported in >10% of patients in either group. *Adverse events were coded based on single preferred terms in the Medical Dictionary for Regulatory Activities (MedDRA) version 22.0, except for abdominal pain, which was based on a group of MedDRA preferred terms. ^†^Based on MedDRA preferred term “interstitial lung disease”. ^‡^Adverse event that resulted in death, was life-threatening, resulted in hospitalization or prolongation of hospitalization, resulted in persistent or clinically significant disability or incapacity, was a congenital anomaly or birth defect, or was deemed to be serious for any other reason. ILD, interstitial lung disease. ALT, alanine aminotransferase; AST, aspartate aminotransferase.
