# Supplementary material for: Safety and tolerability of nintedanib in patients with progressive fibrosing interstitial lung diseases: data from the randomized controlled INBUILD trial
Source: Respir Res. 2022 Apr 7;23:85. doi: 10.1186/s12931-022-01974-2 (PMC8991727; doi:10.1186/s12931-022-01974-2)
Supplement: Supplementary file 5 — Additional file 5: Table S4. Most frequent adverse events in the INBUILD trial in subgroups by sex. Table S5. Most frequent adverse events in the INBUILD trial in subgroups by age at baseline. Table S6. Most frequent adverse events in the INBUILD trial in subgroups by race. Table S7. Most frequent adverse events in the INBUILD trial in subgroups by weight at baseline. [file 12931_2022_1974_MOESM5_ESM.docx]

**Additional file 5: Tables S4-S7**

**Table S4:** Most frequent adverse events in the INBUILD trial in subgroups by sex.

|  | **Male** | | | | **Female** | | | |
| --- | --- | --- | --- | --- | --- | --- | --- | --- |
|  | **Nintedanib (n=179)** | | **Placebo (n=177)** | | **Nintedanib (n=153)** | | **Placebo (n=154)** | |
|  | **n (%)** | **Rate per 100 patient–years** | **n (%)** | **Rate per 100 patient–years** | **n (%)** | **Rate per 100 patient–years** | **n (%)** | **Rate per 100 patient–years** |
| Diarrhea | 132 (73.7) | 133.8 | 47 (26.6) | 24.1 | 108 (70.6) | 139.8 | 38 (24.7) | 21.8 |
| Nausea | 39 (21.8) | 20.1 | 14 (7.9) | 5.9 | 61 (39.9) | 46.5 | 19 (12.3) | 9.5 |
| Vomiting | 20 (11.2) | 9.2 | 8 (4.5) | 3.3 | 44 (28.8) | 28.6 | 8 (5.2) | 3.7 |
| Abdominal pain | 31 (17.3) | 14.8 | 8 (4.5) | 3.3 | 31 (20.3) | 19.2 | 11 (7.1) | 5.2 |
| Nasopharyngitis | 27 (15.1) | 12.7 | 30 (16.9) | 13.6 | 27 (17.6) | 15.4 | 18 (11.7) | 9.0 |
| Decreased appetite | 27 (15.1) | 12.6 | 13 (7.3) | 5.4 | 27 (17.6) | 15.7 | 10 (6.5) | 4.7 |
| Dyspnea | 37 (20.7) | 17.5 | 31 (17.5) | 13.5 | 15 (9.8) | 7.9 | 26 (16.9) | 13.0 |
| Bronchitis | 25 (14.0) | 11.8 | 31 (17.5) | 13.8 | 23 (15.0) | 12.6 | 33 (21.4) | 17.4 |
| Weight decreased | 27 (15.1) | 12.5 | 9 (5.1) | 3.7 | 22 (14.4) | 12.3 | 9 (5.8) | 4.2 |
| ALT increased | 12 (6.7) | 5.2 | 5 (2.8) | 2.0 | 37 (24.2) | 22.3 | 8 (5.2) | 3.8 |
| AST increased | 13 (7.3) | 5.7 | 6 (3.4) | 2.4 | 30 (19.6) | 17.5 | 7 (4.5) | 3.3 |
| Cough | 25 (14.0) | 11.5 | 25 (14.1) | 11.0 | 15 (9.8) | 7.9 | 26 (16.9) | 13.3 |
| Progression of ILD* | 17 (9.5) | 7.3 | 34 (19.2) | 14.8 | 11 (7.2) | 5.6 | 22 (14.3) | 10.5 |

Data are based on adverse events reported between first trial drug intake and 28 days after last trial drug intake. Adverse events were coded based on single preferred terms in the Medical Dictionary for Regulatory Activities (MedDRA) version 22.0, except for abdominal pain, which was based on a group of MedDRA preferred terms. Adverse events with a rate >10 events per 100 patient-years in either treatment group in the overall population are shown. *Based on MedDRA preferred term “interstitial lung disease”. ALT, alanine aminotransferase; AST, aspartate aminotransferase.

**Table S5:** Most frequent adverse events in the INBUILD trial in subgroups by age at baseline.

|  | **<65 years** | | | | **≥65 years** | | | |
| --- | --- | --- | --- | --- | --- | --- | --- | --- |
|  | **Nintedanib (n=139)** | | **Placebo (n=121)** | | **Nintedanib (n=193)** | | **Placebo (n=210)** | |
|  | **n (%)** | **Rate per 100 patient–years** | **n (%)** | **Rate per 100 patient–years** | **n (%)** | **Rate per 100 patient–years** | **n (%)** | **Rate per 100 patient–years** |
| Diarrhea | 99 (71.2) | 123.6 | 25 (20.7) | 17.4 | 141 (73.1) | 147.2 | 60 (28.6) | 26.5 |
| Nausea | 42 (30.2) | 29.8 | 11 (9.1) | 6.7 | 58 (30.1) | 31.5 | 22 (10.5) | 8.1 |
| Vomiting | 24 (17.3) | 14.3 | 5 (4.1) | 2.9 | 40 (20.7) | 19.8 | 11 (5.2) | 3.9 |
| Abdominal pain | 22 (15.8) | 12.9 | 7 (5.8) | 4.2 | 40 (20.7) | 20.0 | 12 (5.7) | 4.2 |
| Nasopharyngitis | 24 (17.3) | 13.9 | 20 (16.5) | 12.9 | 30 (15.5) | 13.8 | 28 (13.3) | 10.6 |
| Decreased appetite | 22 (15.8) | 13.0 | 2 (1.7) | 1.1 | 32 (16.6) | 14.7 | 21 (10.0) | 7.5 |
| Dyspnea | 20 (14.4) | 11.0 | 15 (12.4) | 9.0 | 32 (16.6) | 14.6 | 42 (20.0) | 15.9 |
| Bronchitis | 26 (18.7) | 15.1 | 18 (14.9) | 11.2 | 22 (11.4) | 9.9 | 46 (21.9) | 18.0 |
| Weight decreased | 19 (13.7) | 10.9 | 2 (1.7) | 1.1 | 30 (15.5) | 13.6 | 16 (7.6) | 5.6 |
| ALT increased | 23 (16.5) | 13.4 | 8 (6.6) | 4.7 | 26 (13.5) | 11.6 | 5 (2.4) | 1.7 |
| AST increased | 17 (12.2) | 9.7 | 8 (6.6) | 4.7 | 26 (13.5) | 11.6 | 5 (2.4) | 1.7 |
| Cough | 18 (12.9) | 9.9 | 23 (19.0) | 15.0 | 22 (11.4) | 9.8 | 28 (13.3) | 10.4 |
| Progression of ILD* | 5 (3.6) | 2.6 | 19 (15.7) | 11.5 | 23 (11.9) | 9.8 | 37 (17.6) | 13.5 |

Data are based on adverse events reported between first trial drug intake and 28 days after last trial drug intake. Adverse events were coded based on single preferred terms in the Medical Dictionary for Regulatory Activities (MedDRA) version 22.0, except for abdominal pain, which was based on a group of MedDRA preferred terms. Adverse events with a rate >10 events per 100 patient-years in either treatment group in the overall population are shown. *Based on MedDRA preferred term “interstitial lung disease”. ALT, alanine aminotransferase; AST, aspartate aminotransferase.

**Table S6:** Most frequent adverse events in the INBUILD trial in subgroups by race.

|  | **White** | | | | **Asian** | | | | **Black/African-American** | | | |
| --- | --- | --- | --- | --- | --- | --- | --- | --- | --- | --- | --- | --- |
|  | **Nintedanib (n=242)** | | **Placebo (n=246)** | | **Nintedanib (n=84)** | | **Placebo (n=80)** | | **Nintedanib (n=5)** | | **Placebo (n=5)** | |
|  | **n (%)** | **Rate per 100 patient–years** | **n (%)** | **Rate per 100 patient–years** | **n (%)** | **Rate per 100 patient–years** | **n (%)** | **Rate per 100 patient–years** | **n (%)** | **Rate per 100 patient–years** | **n (%)** | **Rate per 100 patient–years** |
| Diarrhea | 170 (70.2) | 121.6 | 59 (24.0) | 21.1 | 66 (78.6) | 194.1 | 24 (30.0) | 28.1 | 3 (60.0) | 144.8 | 2 (40.0) | 41.3 |
| Nausea | 66 (27.3) | 27.4 | 28 (11.4) | 8.7 | 30 (35.7) | 36.9 | 4 (5.0) | 3.7 | 3 (60.0) | 95.4 | 1 (20.0) | 17.5 |
| Vomiting | 49 (20.2) | 18.6 | 13 (5.3) | 3.8 | 13 (15.5) | 12.6 | 3 (3.8) | 2.7 | 2 (40.0) | 76.8 | 0 | 0 |
| Abdominal pain | 49 (20.2) | 18.6 | 15 (6.1) | 4.4 | 13 (15.5) | 12.8 | 4 (5.0) | 3.6 | 0 | 0 | 0 | 0 |
| Nasopharyngitis | 32 (13.2) | 11.0 | 24 (9.8) | 7.4 | 22 (26.2) | 23.6 | 23 (28.8) | 25.2 | 0 | 0 | 1 (20.0) | 16.0 |
| Decreased appetite | 33 (13.6) | 11.5 | 14 (5.7) | 4.1 | 20 (23.8) | 21.1 | 8 (10.0) | 7.3 | 1 (20.0) | 30.3 | 1 (20.0) | 16.0 |
| Dyspnea | 46 (19.0) | 16.3 | 50 (20.3) | 16.0 | 6 (7.1) | 5.3 | 5 (6.3) | 4.4 | 0 | 0 | 2 (40.0) | 37.2 |
| Bronchitis | 38 (15.7) | 13.4 | 56 (22.8) | 18.7 | 10 (11.9) | 9.4 | 8 (10.0) | 7.4 | 0 | 0 | 0 | 0 |
| Weight decreased | 31 (12.8) | 10.7 | 11 (4.5) | 3.2 | 18 (21.4) | 18.0 | 7 (8.8) | 6.4 | 0 | 0 | 0 | 0 |
| ALT increased | 31 (12.8) | 10.5 | 7 (2.8) | 2.0 | 18 (21.4) | 19.3 | 4 (5.0) | 3.7 | 0 | 0 | 2 (40.0) | 31.6 |
| AST increased | 26 (10.7) | 8.6 | 8 (3.3) | 2.3 | 17 (20.2) | 18.4 | 3 (3.8) | 2.7 | 0 | 0 | 2 (40.0) | 31.6 |
| Cough | 36 (14.9) | 12.5 | 45 (18.3) | 14.7 | 3 (3.6) | 2.6 | 4 (5.0) | 3.6 | 1 (20.0) | 30.8 | 2 (40.0) | 36.0 |
| Progression of ILD* | 13 (5.4) | 4.1 | 27 (11.0) | 8.1 | 15 (17.9) | 13.9 | 29 (36.3) | 29.8 | 0 | 0 | 0 | 0 |

Data are based on adverse events reported between first trial drug intake and 28 days after last trial drug intake. Adverse events were coded based on single preferred terms in the Medical Dictionary for Regulatory Activities (MedDRA) version 22.0, except for abdominal pain, which was based on a group of MedDRA preferred terms. Adverse events with a rate >10 events per 100 patient-years in either treatment group in the overall population are shown. *Based on MedDRA preferred term “interstitial lung disease”. ALT, alanine aminotransferase; AST, aspartate aminotransferase.

**Table S7:** Most frequent adverse events in the INBUILD trial in subgroups by weight at baseline.

|  | **≤65 kg** | | | | **>65 kg** | | | |
| --- | --- | --- | --- | --- | --- | --- | --- | --- |
|  | **Nintedanib (n=91)** | | **Placebo (n=89)** | | **Nintedanib (n=241)** | | **Placebo (n=242)** | |
|  | **n (%)** | **Rate per 100 patient–years** | **n (%)** | **Rate per 100 patient–years** | **n (%)** | **Rate per 100 patient–years** | **n (%)** | **Rate per 100 patient–years** |
| Diarrhea | 67 (73.6) | 173.8 | 25 (28.1) | 26.6 | 173 (71.8) | 126.0 | 60 (24.8) | 21.7 |
| Nausea | 33 (36.3) | 40.8 | 8 (9.0) | 6.9 | 67 (27.8) | 27.5 | 25 (10.3) | 7.8 |
| Vomiting | 22 (24.2) | 23.2 | 3 (3.4) | 2.5 | 42 (17.4) | 15.2 | 13 (5.4) | 3.9 |
| Abdominal pain | 14 (15.4) | 14.8 | 3 (3.4) | 2.4 | 48 (19.9) | 17.3 | 16 (6.6) | 4.8 |
| Nasopharyngitis | 16 (17.6) | 16.9 | 16 (18.0) | 15.0 | 38 (15.8) | 12.9 | 32 (13.2) | 10.2 |
| Decreased appetite | 21 (23.1) | 22.8 | 7 (7.9) | 5.8 | 33 (13.7) | 11.2 | 16 (6.6) | 4.8 |
| Dyspnea | 8 (8.8) | 7.5 | 11 (12.4) | 9.4 | 44 (18.3) | 14.9 | 46 (19.0) | 14.7 |
| Bronchitis | 10 (11.0) | 9.7 | 14 (15.7) | 12.6 | 38 (15.8) | 13.0 | 50 (20.7) | 16.4 |
| Weight decreased | 17 (18.7) | 17.2 | 6 (6.7) | 5.0 | 32 (13.3) | 10.8 | 12 (5.0) | 3.5 |
| ALT increased | 21 (23.1) | 23.3 | 3 (3.4) | 2.5 | 28 (11.6) | 9.2 | 10 (4.1) | 3.0 |
| AST increased | 19 (20.9) | 21.2 | 3 (3.4) | 2.5 | 24 (10.0) | 7.8 | 10 (4.1) | 2.9 |
| Cough | 5 (5.5) | 4.6 | 8 (9.0) | 6.9 | 35 (14.5) | 11.8 | 43 (17.8) | 14.1 |
| Progression of ILD* | 9 (9.9) | 8.3 | 22 (24.7) | 19.9 | 19 (7.9) | 5.9 | 34 (14.0) | 10.3 |

Data are based on adverse events reported between first trial drug intake and 28 days after last trial drug intake. Adverse events were coded based on single preferred terms in the Medical Dictionary for Regulatory Activities (MedDRA) version 22.0, except for abdominal pain, which was based on a group of MedDRA preferred terms. Adverse events with a rate >10 events per 100 patient-years in either treatment group in the overall population are shown. *Based on MedDRA preferred term “interstitial lung disease”. ALT, alanine aminotransferase; AST, aspartate aminotransferase.
