# Supplementary material for: Safety and tolerability of nintedanib in patients with progressive fibrosing interstitial lung diseases: data from the randomized controlled INBUILD trial
Source: Respir Res. 2022 Apr 7;23:85. doi: 10.1186/s12931-022-01974-2 (PMC8991727; doi:10.1186/s12931-022-01974-2)
Supplement: Supplementary file 6 — Additional file 6: Table S8. Most frequent serious adverse events in the INBUILD trial. [file 12931_2022_1974_MOESM6_ESM.docx]

**Additional file 6: Table S8**

Most frequent serious adverse events in the INBUILD trial.

|  | **Nintedanib (n=332)** | | **Placebo (n=331)** | |
| --- | --- | --- | --- | --- |
|  | **n (%)** | **Rate per 100 patient–years** | **n (%)** | **Rate per 100 patient–years** |
| Pneumonia | 24 (7.2) | 5.6 | 16 (4.8) | 3.5 |
| Progression of ILD* | 19 (5.7) | 4.4 | 45 (13.6) | 10.1 |
| Acute respiratory failure | 16 (4.8) | 3.7 | 7 (2.1) | 1.5 |
| Respiratory failure | 11 (3.3) | 2.5 | 10 (3.0) | 2.1 |
| Pulmonary fibrosis | 7 (2.1) | 1.6 | 5 (1.5) | 1.1 |
| Dyspnea | 6 (1.8) | 1.4 | 13 (3.9) | 2.8 |
| Pneumothorax | 6 (1.8) | 1.4 | 6 (1.8) | 1.3 |
| Drug-induced liver injury | 6 (1.8) | 1.4 | 0 | 0 |
| Pulmonary hypertension | 5 (1.5) | 1.1 | 9 (2.7) | 1.9 |
| Atrial fibrillation | 5 (1.5) | 1.1 | 1 (0.3) | 0.2 |
| Bronchitis | 4 (1.2) | 0.9 | 5 (1.5) | 1.1 |
| Chronic respiratory failure | 1 (0.3) | 0.2 | 6 (1.8) | 1.3 |
| Pulmonary embolism | 1 (0.3) | 0.2 | 5 (1.5) | 1.1 |

Data are based on adverse events reported between first trial drug intake and 28 days after last trial drug intake. Median exposure to trial drug was 17.4 months in both groups. Adverse events were coded based on single preferred terms in the Medical Dictionary for Regulatory Activities (MedDRA) version 22.0. Serious adverse events with a rate of >1 event per 100 patient-years in either treatment group are shown. *Based on MedDRA preferred term “interstitial lung disease”.
