# Supplementary material for: Safety and tolerability of nintedanib in patients with progressive fibrosing interstitial lung diseases: data from the randomized controlled INBUILD trial
Source: Respir Res. 2022 Apr 7;23:85. doi: 10.1186/s12931-022-01974-2 (PMC8991727; doi:10.1186/s12931-022-01974-2)
Supplement: Supplementary file 7 — Additional file 7: Table S9. Bleeding adverse events in the INBUILD trial. [file 12931_2022_1974_MOESM7_ESM.docx]

**Additional file 7: Table S9**

Bleeding adverse events in the INBUILD trial

|  | **Nintedanib**  **(n=332)** | | **Placebo**  **(n=331)** | |
| --- | --- | --- | --- | --- |
|  | **n (%)** | **Rate per 100 patient–years** | **n (%)** | **Rate per 100 patient–years** |
| Any bleeding adverse event(s)* | 46 (13.9) | 11.6 | 51 (15.4) | 11.7 |
| Most frequent bleeding adverse events^†^ |  |  |  |  |
| Epistaxis | 14 (4.2) | 3.3 | 11 (3.3) | 2.4 |
| Contusion | 5 (1.5) | 1.1 | 10 (3.0) | 2.2 |
| Hemoptysis | 4 (1.2) | 0.9 | 8 (2.4) | 1.7 |
| Any serious bleeding adverse event(s)*^‡^ | 5 (1.5) | 1.1 | 7 (2.1) | 1.5 |
| Most frequent serious bleeding adverse events^§^ |  |  |  |  |
| Gastrointestinal hemorrhage | 3 (0.9) | 0.7 | 0 | 0 |

Data are n (%) of patients with ≥1 such adverse event reported between first trial drug intake and 28 days after last trial drug intake. Median exposure to trial drug was 17.4 months in both groups. *Based on standardized Medical Dictionary for Regulatory Activities (MedDRA) query “hemorrhage terms (excluding laboratory terms)” (narrow definition). ^†^Based on single MedDRA preferred terms. Events with a rate >1.5 events per 100 patient-years in either treatment group are shown. ^‡^A serious adverse event was defined as an adverse event that resulted in death, was life-threatening, resulted in hospitalization or prolongation of hospitalization, resulted in persistent or clinically significant disability or incapacity, was a congenital anomaly or birth defect, or was deemed to be serious for any other reason. ^§^Based on single MedDRA preferred terms. Events with a rate >0.5 events per 100 patient-years in either treatment group are shown.
