# Supplementary material for: Safety and tolerability of nintedanib in patients with progressive fibrosing interstitial lung diseases: data from the randomized controlled INBUILD trial
Source: Respir Res. 2022 Apr 7;23:85. doi: 10.1186/s12931-022-01974-2 (PMC8991727; doi:10.1186/s12931-022-01974-2)
Supplement: Supplementary file 8 — Additional file 8: Table S10. Cardiovascular events in the INBUILD trial. [file 12931_2022_1974_MOESM8_ESM.docx]

**Additional file 8: Table S10**

Cardiovascular events in the INBUILD trial.

|  | **Nintedanib**  **(n=332)** | | **Placebo**  **(n=331)** | |
| --- | --- | --- | --- | --- |
|  | **n (%)** | **Rate per 100 patient–years** | **n (%)** | **Rate per 100 patient–years** |
| Major adverse cardiovascular events* | 21 (6.3) | 4.9 | 17 (5.1) | 3.7 |
| Hypertension^†^ | 19 (5.7) | 4.5 | 23 (6.9) | 5.1 |
| Cardiac failure^†^ | 7 (2.1) | 1.6 | 7 (2.1) | 1.5 |
| Arterial thromboembolism^†^ | 6 (1.8) | 1.4 | 8 (2.4) | 1.7 |
| Myocardial infarction^†^ | 6 (1.8) | 1.4 | 5 (1.5) | 1.1 |
| Venous thromboembolism^†^ | 3 (0.9) | 0.7 | 8 (2.4) | 1.7 |
| Stroke ischemic and hemorrhagic^†^ | 3 (0.9) | 0.7 | 7 (2.1) | 1.5 |
| Deep vein thrombosis^‡^ | 2 (0.6) | 0.5 | 2 (0.6) | 0.4 |
| Pulmonary embolism^‡^ | 1 (0.3) | 0.2 | 5 (1.5) | 1.1 |

Data are n (%) of patients with ≥1 such adverse event reported between first trial drug intake and 28 days after last trial drug intake. Median exposure to trial drug was 17.4 months in both groups. *Based on fatal adverse events in the MedDRA system organ classes “cardiac disorders” and “vascular disorders”; any fatal and non-fatal events in the subordinate standardized MedDRA query (SMQ) “myocardial infarction” (broad); any fatal and non-fatal stroke events (based on selected MedDRA preferred terms); and the MedDRA preferred terms “sudden death”, “cardiac death” and “sudden cardiac death”. ^†^Based on group of MedDRA preferred terms. ^‡^Based on MedDRA preferred term.
